# Efficacy of folic acid supplementation on endothelial function and plasma homocysteine concentration in coronary artery disease: A meta-analysis of randomized controlled trials

**DOI:** 10.3892/etm.2014.1553

**Published:** 2014-02-17

**Authors:** XIN YI, YANLI ZHOU, DINGSHENG JIANG, XIAOYAN LI, YI GUO, XUEJUN JIANG

**Affiliations:** 1 Department of Cardiology, Renmin Hospital of Wuhan University, Wuhan, Hubei 430060, P.R. China; 2 Cardiovascular Research Institute of Wuhan University, Wuhan, Hubei 430060, P.R. China; 3 Department of Epidemiology, School of Public Health of Wuhan University, Wuhan, Hubei 430060, P.R. China

**Keywords:** folic acid, endothelial function, homocysteine, coronary artery disease, meta-analysis

## Abstract

The aim of the present study was to conduct an updated meta-analysis of relevant randomized controlled trials (RCTs) in order to estimate the effect of folic acid supplementation on endothelial function and the concentration of plasma homocysteine in patients with coronary artery disease (CAD). An extensive search of PubMed was conducted to identify RCTs that compared folic acid with placebo therapy. The mean difference (MD) and 95% confidence interval (CI) were used as a measure of the correlation between folic acid supplementation and endothelial function/plasma homocysteine concentration. Of the 377 patients included in this analysis, 191 patients underwent folic acid supplementation and 186 individuals underwent placebo treatment. Compared with the use of a placebo, folic acid supplementation alone exhibited significant efficacy on increasing flow-mediated dilation (FMD; MD, 57.72 μm; 95% CI, 50.14–65.31; P<0.05) and lowering the concentration of plasma homocysteine (MD, −3.66 μmol/l; 95% CI, −5.44–−1.87; P<0.05; I^2^, 87%). There was no significant change in the response to end diastolic diameter, glyceryl-trinitrate diameter, heart rate, baseline and peak hyperemic flow and systolic and diastolic blood pressure between the folic acid and placebo groups (P>0.05). Therefore, the meta-analysis indicated that 5 mg folic acid daily supplementation for >4 weeks significantly improved FMD and lowered the concentration of plasma homocysteine in patients with CAD. However, more RCTs are required in order to confirm these observations.

## Introduction

Endothelial dysfunction is closely associated with the occurrence and development of atherosclerotic disease and numerous studies have confirmed that coronary artery disease (CAD) is often accompanied by endothelial dysfunction (1,2). A number of studies have demonstrated that hyperhomocysteinemia, one of the risk factors of CAD, promotes the occurrence and development of CAD by damaging vascular endothelial function (3–5). Experimental studies (6–8) and epidemiological data (9) have demonstrated that combined folic acid (400 μg–5 mg daily) and vitamin B therapy may be involved in the regulation of vascular endothelial structure and function. However, there is no definitive conclusion with regard to this effect lowering the concentration of plasma homocysteine. Whether long-term high-dose folic acid (5 mg daily) alone may effectively improve vascular endothelial function and lower the concentration of plasma homocysteine in patients with CAD remains controversial (10–12). Therefore, a meta-analysis of randomized controlled trials (RCTs) with regard to folic acid treatment for CAD was performed to verify whether folic acid is capable of improving endothelial function and reducing the concentration of plasma homocysteine in patients with CAD.

## Materials and methods

### Search strategy for RCTs

**Figure 1:**
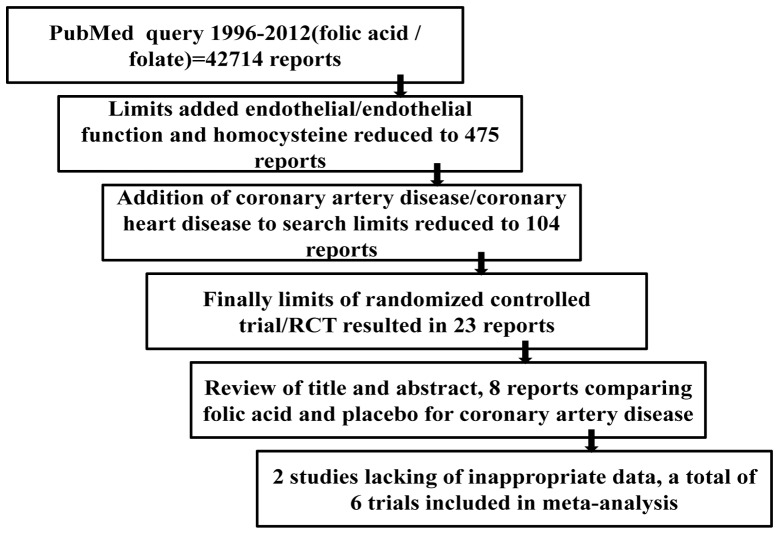
Search process for inclusion in this meta-analysis comparing the efficacy of folic acid supplementation on endothelial function and plasma homocysteine concentration in patients with CAD. CAD, coronary artery disease.

In order to compare the efficacy of folic acid supplementation or placebos for CAD, an extensive literature search on PubMed was performed in order to locate relevant RCTs that were published between January 1966 and September 2012. A flow chart of the search methodology used in this meta-analysis is provided in Fig. 1. A total of 42,714 reports were identified using a PubMed query of ‘folic acid’ or ‘folate’. Limits of ‘endothelial/endothelial function’ and ‘homocysteine’ reduced the number of reports to 475. Further refinement of the search criteria with the addition of ‘coronary artery disease/coronary heart disease’ reduced the number of reports to 104. Finally, limits of ‘randomized controlled trial/RCT’ resulted in a total of 23 reports (6,13–31). The titles and abstracts of the reports were reviewed for terms, including folic acid or folate, endothelial function, CAD or coronary heart disease. Following careful review, eight randomized studies were identified that discussed folic acid and placebo treatment of CAD. However, two studies (10,28) were excluded for their imprecise information with regard to flow-mediated dilation (FMD) and end diastolic diameter (EDD) in the folic acid and placebo-treated groups. As a result, a total of six trials (11,12,17,25,27,31) were used for this meta-analysis. The studies were reviewed by two independent authors in order to assess their quality. Any discrepancies in their judgments were resolved by joint discussion or discussion with a third reviewer, referencing the original report. Variable trials that were assessed included an accurate description of methods including study design, inclusion criteria, exclusion criteria, the statistical tests used, the baselines between the patients undergoing folic acid or placebo treatment and the outcome of the measures reported along with the results of the follow-up.

### Inclusion/exclusion criteria

Studies were eligible for inclusion if they met the following criteria: i) the study was a RCT; ii) the study was conducted using human subjects with CAD; iii) active treatment consisted of folic acid supplementation (without additional vitamin B supplementation); iv) folic acid was administered orally with a dose of 5 mg/day; v) the duration of active treatment was ≥4 weeks and ≤16 weeks; vi) plasma homocysteine concentration was provided; vii) the study reported the mean FMD and/or EDD for the treatment and placebo groups. Studies that reported either FMD or EDD changes alone, assuming all other criteria were met, were included in this meta-analysis.

Although 23 potentially relevant studies were identified and screened, 17 trials did not meet the inclusion criteria for this meta-analysis. Major reasons for the exclusion of a study were i) the patients were also treated with vitamin B; ii) the subject populations did not have CAD; iii) the dosage of folic acid was not 5 mg/day; iv) the trials were not randomized; v) there was an absence of data by which to calculate the changes in FMD or EDD.

### Data abstraction and statistical analysis

Information with regard to study design, sample size, duration, clinical characteristics and the medication of the participant, as well as biochemical parameters and treatment results regarding endothelial function, were independently abstracted from the six clinical trials and subsequently entered as standard forms into a Microsoft Excel (Microsoft Corporation, Redmond, Washington, WA, USA) spreadsheet to calculate the overall efficacy following folic acid supplementation compared with that following the administration of a placebo. The risk of bias was assessed as recommended in the Cochrane Handbook by RevMan 5.0 (The Cochrane Collaboration) and the standards of assessment were as follows: i) adequate sequence generation; ii) allocation concealment; iii) incomplete outcome data were addressed; iv) free of selective reporting; v) free of other bias. On the basis of this assessment methodology, the two reviewers provided each eligible study with an overall rating of low, high or an unclear risk of bias. Once the outcomes had been evaluated, a table summarizing the observations was created using the Grading of Recommendations Assessment, Development and Evaluation (GRADE) system.

The data were analyzed according to the intention-to-treat principle. RevMan 5.0 was used for the meta-analysis. The mean difference (MD) with 95% confidence interval (CI) were calculated as a measure of the correlation between folic acid supplementation and endothelial function/plasma homocysteine concentration. A two-sided P<0.05 was considered to indicate a statistically significant difference.

P values from χ^2^ statistical analysis and I^2^ were used for the heterogeneity test. Heterogeneity was considered to be significant when P<0.05. If P>0.1 or I^2^<50%, ‘there may be no heterogeneity among included studies and summarize data across the trials by selecting a fixed-effects model with the software RevMan 5.0’ and pooled data across the trials by selecting a fixed-effects model based on inverse variance methods. Otherwise, the results were considered to have ‘considerable heterogeneity’ and were compared using a random-effects model. Publication bias was assessed by funnel plots with the standard error of the intervention effect on the vertical axis and MD measuring the effect of intervention on the horizontal axis.

## Results

### Characteristics of included RCTs

Participant and study design characteristics for the six RCTs included in the meta-analysis are shown in Table I. Among these clinical studies, four trials had a parallel double-blind design and two had a crossover double-blind design. Of the 377 patients included, 191 patients underwent folic acid supplementation and 186 patients underwent placebo treatment. The majority of trials included aged male participants. Trial duration varied between eight weeks and four months. The medication administered in the variable trials is presented in Table II. There were no differences in the baseline clinical or biochemical parameters (Tables I and II). The majority of patients were treated with antiplatelet therapy, lipid-lowering therapy, β-blockers, angiotensin-converting-enzyme inhibitors, nitrates and other drug therapies. Biochemical parameters, including total cholesterol, triglycerides, low-density lipoprotein (LDL) and high-denstity lipoprotein cholesterol, plasma folic acid, vitamin B_12_, creatinine and glucose, are shown in Table III. Furthermore, baseline endothelial function data of the folic acid and placebo groups are presented in Table IV.

### Assessment of the bias risk and recommended classification of included studies

**Figure 2:**
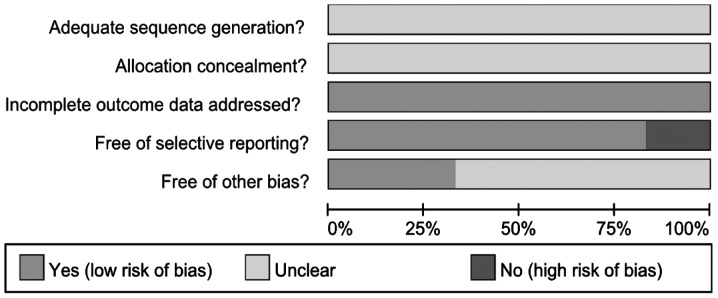
Risk of bias graph. Light grey squares indicate an unclear risk of bias, dark grey squares indicate high risk of bias and medium grey squares indicate low risk of bias.

**Figure 3:**
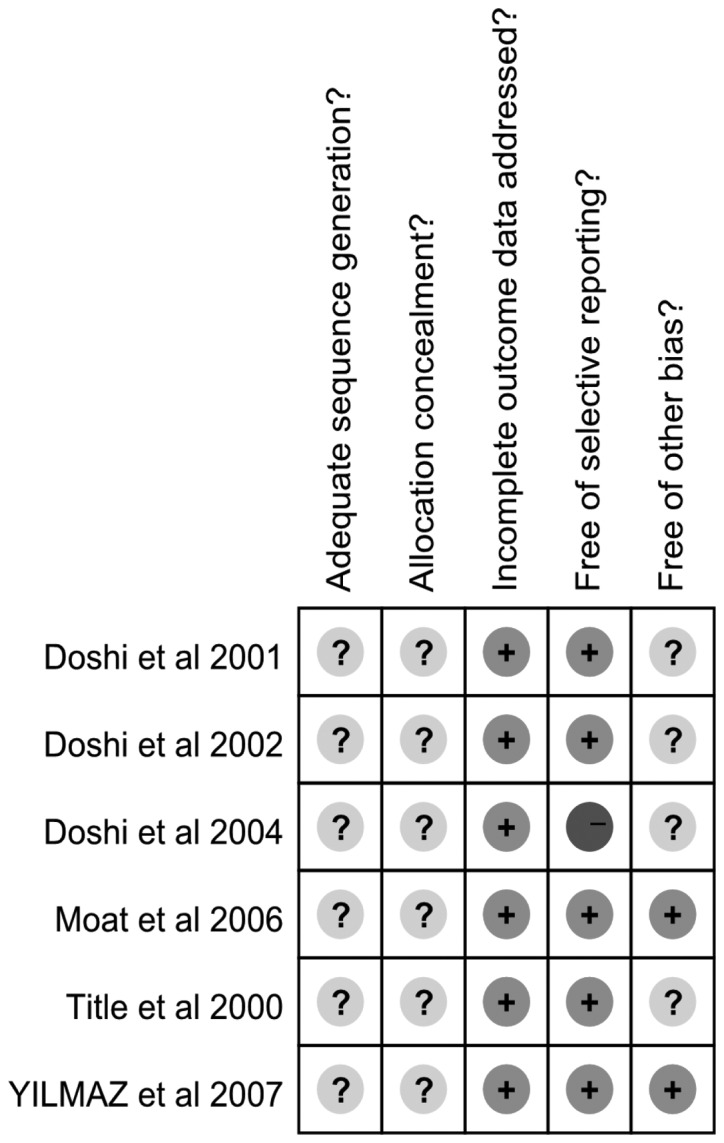
Risk of bias summary. ‘?’ Indicates unclear risk of bias, ‘−’ indicates high risk of bias and ‘+’ indicates a low risk of bias.

The bias risk for the included trials was assessed according to the assessment methodology recommended by The Cochrane Collaboration (Figs. 2 and 3). Adequate sequence generation and allocation concealment were not described clearly in all six studies. The six trials reported complete outcome data, but one study did not clearly describe the selective reporting (12). Due to incomplete information in four studies (12,25,27,31), there may be other biases. The efficacy of folic acid supplementation on FMD and the concentration of plasma homocysteine was the main outcome in this meta-analysis. The recommended classification of FMD was deemed to be of low quality, but the grade of evidence for the concentration of plasma homocysteine was deemed to be of moderate quality. Therefore, due to the quality of evidence according to the GRADE system, the use of folic acid is recommended to reduce the concentration of plasma homocysteine.

### Efficacy of folic acid on endothelial function

**Figure 4:**
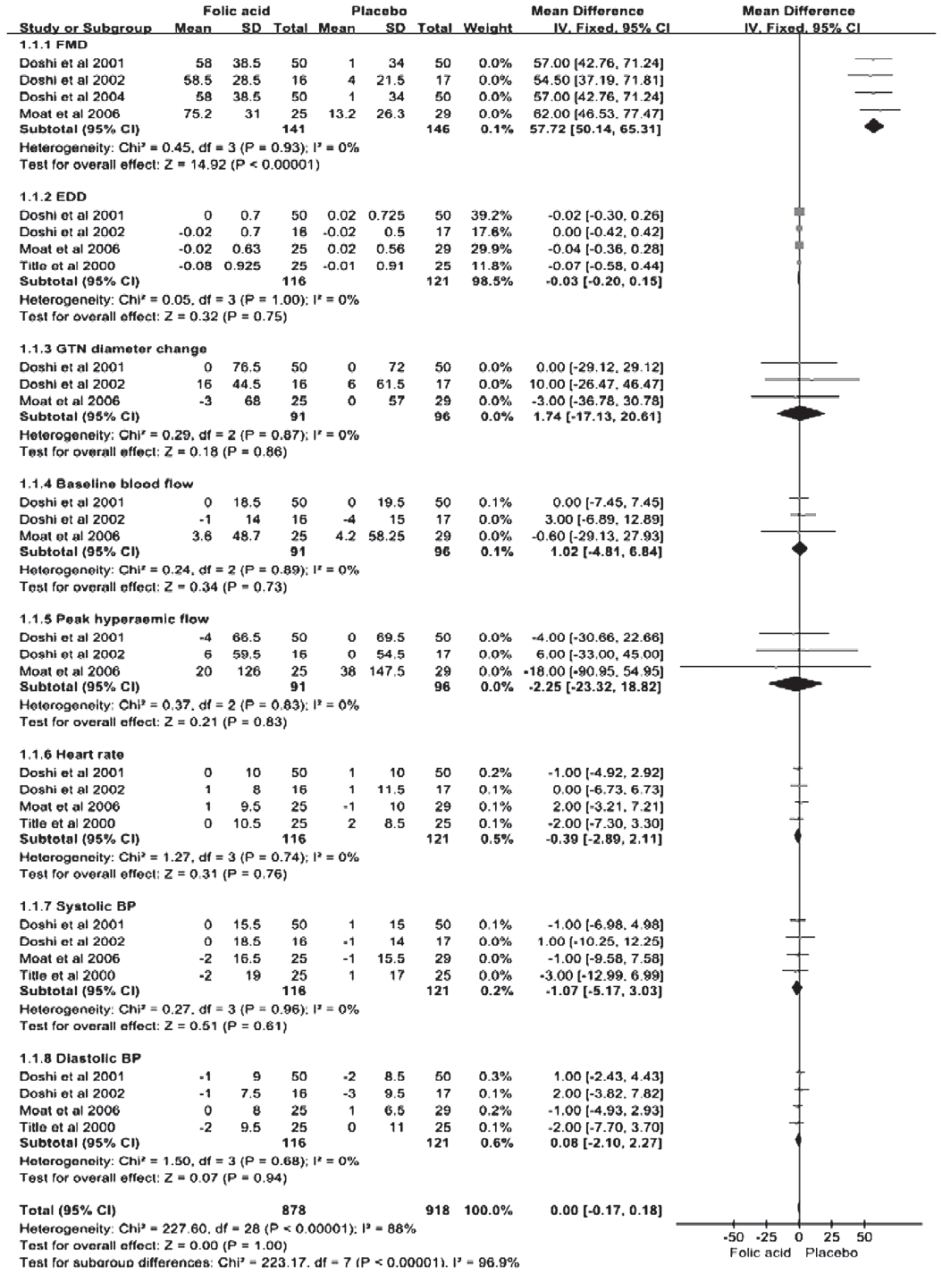
MD with 95% CI estimates for FMD, EDD, GTN diameter change, heart rate, baseline and peak hyperemic flow, systolic and diastolic BP (folic acid vs. placebo), by summarizing different results of included trials in this study. MD, mean difference; CI, confidence interval; FMD, flow-mediated dilation; EDD, end diastolic diameter; GTN, glyceryl-trinitrate; BP, blood pressure.

**Figure 5:**
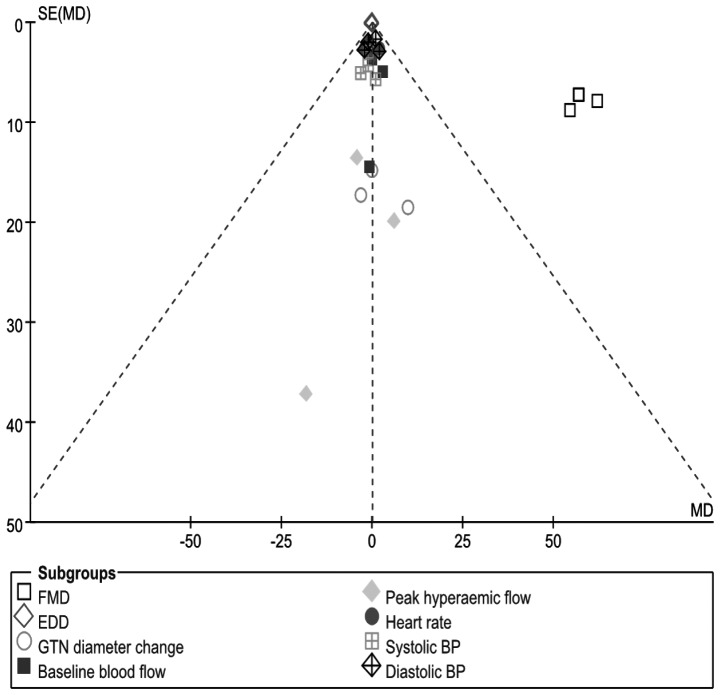
Funnel plot of FMD, EDD, GTN diameter change, heart rate, baseline and peak hyperemic flow, systolic and diastolic BP (folic acid vs. placebo), by summarizing different results of included trials in this study. FMD, flow-mediated dilation; EDD, end diastolic diameter; GTN, glyceryl-trinitrate; BP, blood pressure.

The individual trial results for the effects of folic acid and placebo therapy on FMD, EDD, glyceryl-trinitrate (GTN) diameter change, heart rate, baseline and peak hyperemic flow, systolic and diastolic blood pressure (BP) and the pooled estimate of the effect are shown in Fig. 4. Of the six trials included, four studies measured the efficacy of folic acid on FMD; the pooled estimate from these studies exhibited a marked increase in FMD in the folic acid-treated group when compared with the placebo group (MD, 57.72 μm; 95% CI, 50.14–65.31; P<0.05; I^2^, 0%). Using a random-effects versus a fixed-effects model did not markedly alter the pooled estimate. However, the pooled estimate presented no significant difference in the response to EDD (MD, −0.03; 95% CI, −0.20–0.15; P=0.75; I^2^, 0%), GTN diameter change (MD, 1.74; 95% CI, −17.13–20.61; P=0.86; I^2^, 0%), heart rate (MD, −0.39; 95% CI, −2.89–2.11; P=0.76; I^2^, 0%), baseline hyperemic flow (MD, 1.02; 95% CI, −4.81–6.84; P=0.73; I^2^, 0%), peak hyperemic flow (MD, −2.25; 95% CI, −23.32–18.82; P=0.83; I^2^, 0%), systolic BP (MD, −1.07; 95% CI, −5.71–3.03; P=0.61; I^2^, 0%) and diastolic BP (MD, 0.08, 95% CI, −2.10–2.27; P=0.94; I^2^, 0%) between the folic acid and placebo treatment groups when using a fixed-effects model. A funnel plot of effect size versus study precision was asymmetrical with a relative dearth of moderately precise negative studies, indicating the presence of a positive publication bias (Fig. 5).

### Effect of folic acid on the concentration of plasma homocysteine

**Figure 6:**
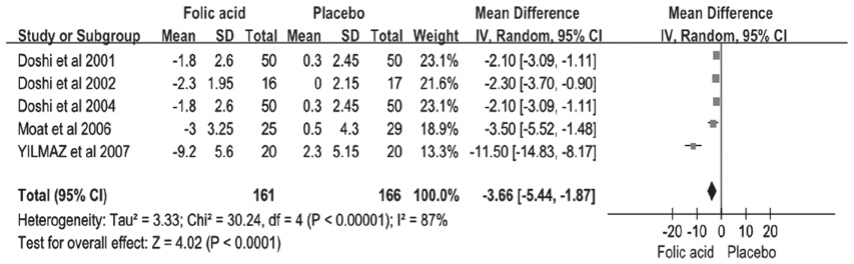
MD with 95% CI estimates for plasma homocysteine concentration (folic acid vs. placebo), by summarizing different results of included trials in this study. MD, mean difference; CI, confidence interval.

In total, five studies reported a change in the concentration of plasma homocysteine (Fig. 6). The results from the random-effects model pooling the MD demonstrated that folic acid supplementation correlated with a significant reduction in the concentration of plasma homocysteine (MD, −3.66 μmol/l; 95% CI, −5.44–−1.87; P<0.05; I^2^, 87%). Using a fixed-effects versus random-effects model did not substantially alter the pooled estimate.

## Discussion

The results of the present meta-analysis demonstrated that an increase in FMD and decrease in plasma homocysteine concentration in CAD patients were associated with folic acid supplementation. However, there was no significant change in EDD, GTN diameter, heart rate, baseline and peak hyperemic flow and systolic and diastolic BP between the folic acid supplementation and placebo-treated groups. Measuring the FMD of a brachial artery using color Doppler ultrasound may accurately reflect the state of coronary endothelial function and serve as a non-invasive method to evaluate endothelial function, thus, is of great value clinically (32,33). Notably, the results of the current RCT meta-analysis are in agreement with data from prospective cohort studies, indicating the efficacy of high-dose folic acid supplementation in improving endothelial function and lowering the concentration of plasma homocysteine in subjects with CAD.

It has been recognized that hyperhomocysteinemia is a risk factor for CAD. Compared with individuals without CAD, the risk of CAD increases 2-fold in patients with hyperhomocysteinemia (34). The function of folic acid is limited to decreasing the levels of plasma cysteine initially, but subsequent studies have demonstrated that folic acid (400 μg/day) can markedly reduce plasma homocysteine levels, while a larger dose of folic acid improves endothelial function in patients with CAD and reduces the incidence of cardiovascular events (11,13). Doshi *et al* (25) observed that an improvement in FMD occurred prior to a significant drop in plasma homocysteine concentration with folic acid treatment, indicating that the enhancement was not explained by a reduction in homocysteine levels. Following the administration of folic acid, FMD improved markedly at 2 h and peaked 4 h after the first dose. However, there was no significant decrease in the total or free plasma homocysteine levels in the 4 h following the initial dose of folic acid. Verhaar *et al* (35) demonstrated that 5-methyltetrahydrofolate, a major circulating folate, is capable of improving endothelial function in patients with familial hypercholesterolemia who are free of vascular disease and are not receiving lipid-lowering treatment. The phenomenon, which may be mediated by an increase in nitric oxide (NO) bioavailability and the generation of superoxide ions, was also confirmed by *in vitro* laboratory experiments (11,35). Schwammenthal *et al* (36), by performing a meta-analysis of a large number of folic acid clinical trials, predicted that folic acid supplementation at dose of 200 μg/day was capable of reducing plasma homocysteine levels by an average of 4 μmol/l. In addition, the authors hypothesized that it may be possible to reduce the number of patients succumbing to cardiovascular disease by 13,500–50,000 each year in the USA. A recent meta-analysis of 12 RCTs using 16,958 subjects found that folic acid supplementation had no efficacy on reducing the risk of CAD (37). However, it should be noted that half of the trials included in this meta-analysis used folic acid dosages that were <5,000 μg/day. An additional meta-analysis (38) observed that the changes in BP and FMD, along with the concomitant changes in the risk of coronary heart disease, may only be observed when folic acid doses are in the order of 5,000 μg/day or greater.

The mechanism by which folic acid improves endothelial function remains unclear, however, previous studies have shown that the phenomenon is likely to be associated with the following mechanisms. Firstly, reduced plasma homocysteine levels. Homocysteine is capable of promoting the generation of hydrogen peroxide and oxygen-derived free radicals by the autoxidation of the sulfhydryl on homocysteine, causing the vascular endothelium to be damaged. This results in abnormal changes to the vascular endothelial cell cytoskeleton, accelerating the oxidation of LDL, increasing the formation of foam cells, thickening the walls of blood vessels and even leading to occlusion of blood vessels. Furthermore, homocysteine may also induce apoptosis in endothelial cells and affect the expression of adhesion factors and cytokines, reducing NO-dependent vasorelaxation (39). However, as mentioned previously, high-dose folic acid supplementation improves endothelial function and reduces plasma homocysteine levels, but does not correlate positively (25). A second potential mechanism is that vascular endothelial cells are weakened by oxidative stress. The biological activity of NO directly affects endothelial function and NO biological activity is determined by the activity of nitric oxide synthase (NOS) and NO inactivation. Various pathophysiological factors are capable of causing the decoupling of eNOS, the result of which produces NO which is converted into generating oxygen-derived free radicals. In recent years, studies have found that the NOS cofactor, tetrahydrobiopterin, is an important regulator of NOS function, which maintains the enzymatic coupling of L-arginine oxidation in order to produce NO (40). NO inactivation is mostly determined by a variety of reactive oxygen-derived free radicals. Thirdly, an additional mechanism may be that folic acid, as a specific type of one-carbon substitution, may be important in repairing genetic damage and maintaining genetic stability (41). Finally, NO production may be directly improved by enhancing the enzymatic activity of eNOS, however, the scavenging potency is 20-fold lower than that of vitamin C (42,43).

Imbalance in the secretion and release of vasoactive substances due to vascular endothelial cell injury leads to spasming of the coronary artery, rupturing of the coronary artery plaque, platelet aggregation and thrombus formation. In addition, it reduces the antithrombotic ability of endothelial cells and increases blood coagulation, which causes thrombosis, ultimately promoting the occurrence and development of CAD. Therefore, improving endothelial function has great clinical value for the prevention and treatment of CAD. The present meta-analysis predicts that the long-term use of high-dose folic acid may reduce the concentration of plasma homocysteine and increase FMD, improving endothelial function. Subsequently, the prevention and treatment of CAD may be achieved via clinical trials of folic acid intervention with CAD patients.

It is possible that high-doses of folic acid (5 mg daily) administered orally improves endothelial function and lowers the concentration of plasma homocysteine in CAD patients. Folic acid supplementation is inexpensive, potentially effective and temporarily devoid of adverse effects. Therefore, folic acid has an exceptionally favorable benefit/risk ratio for improving endothelial function in CAD patients.

There are several limitations to consider when interpreting the results of the present study. Firstly, only two trials included in the meta-analysis truly divided the patients into folic acid supplementation and placebo groups. In the additional fours studies, the patients were randomized to folic acid 5 mg or folic acid 400 μg/N-acetylcysteine/folic acid combined with vitamin B or a placebo. An additional limitation was the small number of cases. No report documented data on the side effects of high dose folic acid and the studies also had markedly different durations and evaluation indices for endothelial function. Thirdly, the current study is prone to the well-known limitation of meta-analyses, namely variation in study design and publication bias. Furthermore, the meta-analytical approach with observational data is even more fraught with limitations. Thus, additional double-blind, randomized, placebo-controlled, multicenter studies with high quality and longer follow-up periods are required to confirm the conclusions of the present study. It will be useful to observe whether the efficacy of folic acid supplementation, particularly on arterial function, is similar among patients with angina, myocardial infarction and non symptom coronary heart disease.

In conclusion, the present meta-analysis of RCTs demonstrates that folic acid supplementation of 5 mg/day for >4 weeks significantly improves FMD and lowers plasma homocysteine concentration in patients with CAD. Thus, this study has underlined the importance of high-dose folic acid supplementation for the improvement of endothelial function. Folic acid supplementation should be recommended for CAD patients. However, more RCTs are required in order to confirm this meta-analysis.

## Figures and Tables

**Table I:** Clinical characteristics of studies included in the meta-analysis.

	Yilmaz *et al* 2007 (17)		Moat *et al* 2006 (11)		Doshi *et al* 2004 (12)		Doshi *et al* 2002 (25)		Doshi *et al* 2001 (27)		Title *et al* 2000 (31)	
						
Characteristics	Placebo	Folic acid	Placebo	Folic acid	Placebo	Folic acid	Placebo	Folic acid	Placebo	Folic acid	Placebo	Folic acid
Study design	PD		PD		CD		PD		CD		PD	
Cases (n)	20	20	29	25	50	50	17	16	50	50	25	25
Age (years)	65.5±7.6	52.2±11.9	61±7	60±7	NA	57±8	56±7	55±7	NA	57±8	60.6±8.6	57.2±9.8
Gender, M/F (n)	18/2	13/7	25/4	21/4	NA	44/6	16/1	14/2	NA	44/6	21/4	19/6
Follow-up (weeks)	8.4±1.1	8.4±1.1	6	6	6	6	6	6	6	6	16	16
BMI (kg/m^2^)	28.3±4	27.2±3.8	29.6±4.1	29.9±4.4	NA	28.5±4.4	NA	NA	NA	28.5±4.4	NA	NA
Diabetes mellitus (n)	8	4	NA	NA	NA	NA	NA	NA	NA	NA	NA	NA
Hypertension (n)	17	13	9	11	NA	NA	7	5	NA	20	11	11
Hyperlipidemia (n)	NA	NA	NA	NA	NA	NA	NA	NA	NA	NA	NA	NA
Myocardial infarction (n)	NA	NA	16	12	NA	33	10	9	NA	33	14	11
Cerebrovascular event (n)	NA	NA	2	1	NA	NA	1	0	NA	4	NA	NA
Smoking (n)	2	6	21	21	NA	NA	13	10	NA	36	6	6
Family history of CAD (n)	7	10	18	12	NA	NA	11	9	NA	26	NA	NA

PD, parallel double-blind; CD, crossover double-blind; M, male; F, female; BMI, body mass index; CAD, coronary artery disease; NA, not available. Data are presented as the mean ± SD.

**Table II:** Clinical medication of studies included in the meta-analysis.

	Yilmaz *et al* 2007 (17)		Moat *et al* 2006 (11)		Doshi *et al* 2004 (12)		Doshi *et al* 2002 (25)		Doshi *et al* 2001 (27)		Title *et al* 2000 (31)	
						
Medication	Placebo	Folic acid	Placebo	Folic acid	Placebo	Folic acid	Placebo	Folic acid	Placebo	Folic acid	Placebo	Folic acid
Aspirin	16	20	NA	NA	NA	46	NA	NA	NA	46	22	20
Clopidogrel	NA	NA	NA	NA	NA	2	NA	NA	NA	2	NA	NA
Nitrates	13	10	6	1	NA	4	2	1	NA	4	NA	NA
β-blockers	10	14	17	16	NA	33	10	6	NA	33	18	21
Statins	14	11	NA	NA	NA	43	14	14	NA	44	NA	NA
ACE inhibitors	8	14	8	9	NA	7	1	1	NA	7	5	9
ATII receptor antagonist	NA	NA	0	1	NA	3	0	1	NA	3	NA	NA
Diuretics	6	5	NA	NA	NA	NA	NA	NA	NA	NA	NA	NA
Calcium channel blocks	0	1	4	3	NA	12	2	3	NA	12	11	5
Insulin	NA	NA	NA	NA	NA	NA	NA	NA	NA	NA	NA	NA
Oral hypoglycemic agent	NA	NA	NA	NA	NA	NA	NA	NA	NA	NA	NA	NA

ACE, angiotensin-converting enzyme; ATII, angiotensin II; NA, not available.

**Table III:** Biochemical parameters of the studies included in the meta-analysis.

	Yilmaz *et al* 2007 (17)		Moat *et al* 2006 (11)		Doshi *et al* 2004 (12)		Doshi *et al* 2002 (25)		Doshi *et al* 2001 (27)		Title *et al* 2000 (31)	
						
Parameters	Placebo	Folic acid	Placebo	Folic acid	Placebo	Folic acid	Placebo	Folic acid	Placebo	Folic acid	Placebo	Folic acid
Homocysteine (μmol/l)												
Baseline	18.4±3.2	21.7±8.7	12.1±3.9	12.9±3.9	10.5±2.5	11.1±2.8	10.8±2.2	10.6±2.6	10.5±2.5	11.1±2.8	12.1	12.3
Follow-up	20.7±7.1	12.5±2.5	12.6±4.7	9.9±2.6	10.8±2.4	9.3±2.4	10.8±2.1	8.3±1.3	10.8±2.4	9.3±2.4	11.8	10.9
Folic acid	12.7±4.1 ng/ml	12.1±5.1 ng/ml	22.7±10.7 nmol/l	20.2±8.6 nmol/l	9.3±2.9 μg/l	8.9±3.5 μg/l	26.09±6.8 nmol/l	22.37±8.7 nmol/l	9.3±2.9 μg/l	8.9±3.5 μg/l	14.7 nmol/l	13.8 nmol/l
Vitamin B_12_	311.9±96.8 pg/ml	296.0±48.7 pg/ml	318±110 pmol/l	366±127 pmol/l	NA	NA	312±105 pmol/l	306±73 pmol/l	430±125.75 ng/l	435±123 ng/l	218 nmol/l	227 nmol/l
Total cholesterol	175.7±51.0 mg/dl	205.2±37.5 mg/dl	4.4±0.6 mmol/l	4.6±0.7 mmol/l	NA	NA	4.47±0.57 mmol/l	4.36±0.87 mmol/l	4.6±0.7 mmol/l	4.8±0.7 mmol/l	5.2 mmol/l	5.3 mmol/l
Triglycerides	108.6±61.1 mg/dl	122.1±39.7 mg/dl	1.77±0.63 mmol/l	1.45±0.69 mmol/l	NA	NA	1.45±0.7 mmol/l	1.43±0.45 mmol/l	1.7±0.9 mmol/l	1.7±0.9 mmol/l	2.5 mmol/l	2.1 mmol/l
LDL	107.6±43.4 mg/dl	128.2±27.2 mg/dl	2.5±0.6 mmol/l	2.7±0.6 mmol/l	NA	NA	2.65±0.48 mmol/l	2.7±0.77 mmol/l	2.8±0.6 mmol/l	2.8±0.6 mmol/l	3.3 mmol/l	3.5 mmol/l
HDL	48.3±9.4 mg/dl	43.6±11.8 mg/dl	1.1±0.2 mmol/l	1.3±0.2 mmol/l	NA	NA	1.16±0.36 mmol/l	1.08±0.2 mmol/l	1.1±0.3 mmol/l	1.2±0.4 mmol/l	0.9 mmol/l	0.9 mmol/l
VLDL (mg/dl)	22.6±11.8	23.5±7.8	NA	NA	NA	NA	NA	NA	NA	NA	NA	NA
Creatinine (μmol/l)	NA	NA	88.1±12.2	89.5±14.0	NA	NA	95.1±12.1	98.7±18.1	98.7±13.6	98.9±13.7	100±19	95±20
HbA1C (%)	NA	NA	NA	NA	NA	NA	NA	NA	NA	NA	NA	NA
Glucose (mmol/l)	NA	NA	5.5±0.7	5.4±0.5	NA	NA	5.49±0.75	5.6±0.77	5.3±0.6	5.3±0.7	NA	NA

LDL, low-density lipoprotein; HDL, high-density lipoprotein; VLDL, very low-density lipoprotein; NA, not available; HbA1C, glycated hemoglobin. Data are presented as the mean ± SD.

**Table IV:** Endothelial function parameters of the studies included in the meta-analysis.

	Yilmaz *et al* 2007 (17)		Moat *et al* 2006 (11)		Doshi *et al* 2004 (12)		Doshi *et al* 2002 (25)		Doshi *et al* 2001 (27)		Title *et al* 2000 (31)	
						
Parameters	Placebo	Folic acid	Placebo	Folic acid	Placebo	Folic acid	Placebo	Folic acid	Placebo	Folic acid	Placebo	Folic acid
FMD (μm)												
Baseline	NA	NA	20.3±31.0	24.4±26.3	46±33	52±34	48±24	52.5±29	46±33	52±34	2.7±3.3%	3.2±3.6%
Follow-up	NA	NA	33.5±21.6	99.6±35.7	47±35	110±43	52±19	111±28	47±35	110±43	2.9±3.7%	5.2±3.9%
EDD												
Baseline	5.8±1.9 %	5.3±2.2 %	4.01±0.52 mm	3.87±0.62 mm	NA	NA	4.20±0.5 mm	4.29±0.7 mm	4.36±0.73 mm	4.39±0.70 mm	4.27±0.91 mm	4.44±0.98 mm
Follow-up	6.1±2.7 %	12.0±6.3 %	4.03±0.6 mm	3.85±0.64 mm	NA	NA	4.18±0.5 mm	4.27±0.7 mm	4.38±0.72 mm	4.39±0.70 mm	4.26±0.91 mm	4.36±0.87 mm
GTN diameter change (μm)												
Baseline	NA	NA	451±55	444±69	NA	NA	396±63	415±52	340±72	340±76	NA	NA
Follow-up	NA	NA	451±59	441±67	NA	NA	402±60	431±37	340±72	340±77	NA	NA
Baseline blood flow (ml/min)												
Baseline	NA	NA	70.4±64.0	70.4±53.1	NA	NA	37±18	35±15	40±20	40±19	NA	NA
Follow-up	NA	NA	74.6±52.5	74.0±44.3	NA	NA	33±12	34±13	40±19	40±18	NA	NA
Peak hyperemic flow (ml/min)												
Baseline	NA	NA	220±135	220±137	NA	NA	189±54	180±67	196±68	202±67	NA	NA
Follow-up	NA	NA	258±160	240±115	NA	NA	189±55	186±52	196±71	198±66	NA	NA
Heart rate (beats/min)												
Baseline	NA	NA	61±10	61±10	NA	NA	61±11	58±8	59±10	59±10	57±10	60±10
Follow-up	NA	NA	60±10	62±9	NA	NA	62±12	59±8	60±10	59±10	59±7	60±11
Systolic BP (mmHg)												
Baseline	135.0±21.9	132.7±21.7	128±15	131±17	NA	NA	131±15	133±18	132±16	133±17	132±17	132±20
Follow-up	NA	NA	127±16	129±16	NA	NA	130±13	133±19	133±14	133±14	133±17	130±18
Diastolic BP (mmHg)												
Baseline	82.7±11.6	82.2±11.7	75±7	77±8	NA	NA	75±10	71±6	73±9	74±9	79±10	81±10
Follow-up	NA	NA	76±6	77±8	NA	NA	72±9	70±9	71±8	73±9	79±12	79±9

FMD, flow-mediated dilatation; EDD, end diastolic diameter; GTN, glyceryl-trinitrate; BP, blood pressure; NA, not available. Data are presented as the mean ± SD.
